# Bilateral cystic lesions of the chest wall: Presentation of scapulothoracic bursitis

**DOI:** 10.1016/j.ijscr.2018.11.035

**Published:** 2018-11-22

**Authors:** Anton Dzian, Michaela Skaličanová, Ivan Fučela, Marek Malík, Jozef Mičák

**Affiliations:** a Department of Thoracic Surgery, Jessenius Faculty of Medicine in Martin, Comenius University in Bratislava, University Hospital in Martin, Kollárova 2, 036 59 Martin, Slovak Republic; b Department of Pathological Anatomy, Jessenius Faculty of Medicine in Martin, Comenius University in Bratislava, University Hospital in Martin, Kollárova 2, 036 59 Martin, Slovak Republic

**Keywords:** Scapulothoracic joint, Bursa, Bursitis, Bilateral resistances

## Abstract

**Introduction:**

Scapulothoracic articulation has several bursae which allow a sliding movement of the scapulothoracic joint. The two major anatomical bursae are the supraserratus bursa and infraserratus bursa.

**Presentation of Case:**

It was a case of a 59 year-old female patient with a professional load and a clinical finding of bilateral subscapular resistances of the thorax. The finding of bilateral collections in the intermuscular spaces between external intercostal muscles and heads of serratus anterior muscle was verified by magnetic resonance.

**Conclusion:**

Due to progressively increasing bilateral findings an open surgical resection was implemented, with good result.

## Highlights

• Rare case of bilateral scapulothoracic bursitis.
• Collections verified by magnetic resonance.
• Open surgical resection with good result.

## 1 Introduction

In the scapulothoracic joint, there are two major and four adventitial bursas. In consequence of mechanical stress, sport or trauma, a bursa inflammation arises: bursitis [1]. The scapulothoracic bursitis manifests itself in patients as pain with increasing activity and a swelling without reddening. By inspection well defined and movable cystic resistance between musculus serratus anterior and the thoracic wall will be revealed. In some patients, a crepitus can be audible and palpable in the movement of the scapula. Magnetic resonance is the best imaging tool for revelation of soft tissue pathology. The treatment of bursitis may be conservative or surgical. The surgical therapy is indicated if conservative treatment is unsuccessful. Open bursectomy, partial scapulectomy and resection of bursa by mini-invasive arthroscopic technique are the most frequent procedures [2]. In the following case the authors describe the case of a female patient with rare bilateral manifestation of tactile resistances in subscapular regions of the thoracic wall.

This case report has been reported in line with the SCARE criteria.

## 2 Case

A 59 year-old female patient, who has been employed as rehabilitation worker, has observed gradually enlarging formations under both her right and left scapula for approximately eight months. In anteflexion, elevation of the upper extremities and when stretching the arms forward, swellings reaching up to the rear axillary lines appear bilaterally subscapularly. They were of soft consistency at palpation. The patient also described pain in the upper extremities, and in the region of arms. She had no recollection of any accident or fall. However, she had undergone neurosurgical operating procedures of disc extrusion in the cervical and thoracic spine, and the findings of bilateral resistances were present already pre-operatively. In the another surgical workplace repeated punctures and partial resection of the swelling on the right side were implemented 5 months ago, and it came to its subsequent recurrence.

**Fig. 1:**
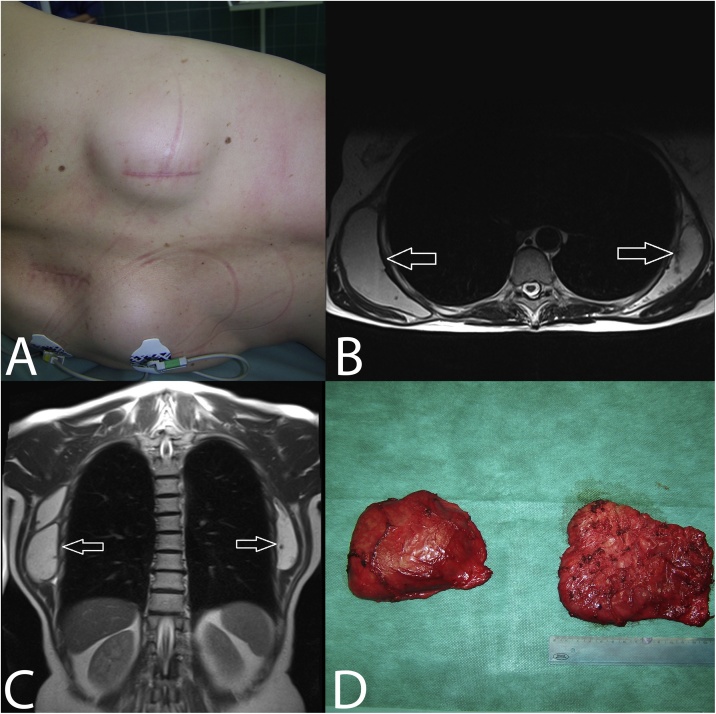
(a): Clinical finding of bilaterally subscapular masses of soft consistency at palpation reaching up to posterior axillary line. (b), (c): Magnetic resonance of thorax displays in dorsolateral parts in subscapular regions symmetrical limited fluid collections with dimensions of 120 × 37 × 115 mm with a volume of 250 ml on the right side and on the left side of 120 × 24 × 90 mm with a volume of 130 ml. The collections are localised in intermuscular spaces between musculi serrati anteriores and external intercostal muscles. (d): Operative findings of encapsulated cystic formations separated from muscles and thorax wall, subsequently sent for histological examination.

The magnetic resonance (Fig. 1(b) and (c)) on thoracic wall showed in dorsolateral parts in subscapular regions in the level of 3rd to 7th rib symmetrical limited fluid collections with dimensions of 120 × 37 x 115 mm on the right side with a volume of 250 ml and on the left side 120 × 24 x 90 mm with a volume of 130 ml. The collections were localised in the intermuscular spaces between the external intercostal muscles and the heads of the muscle serratus anterior. The contents of collections were moderately heterogeneous with sporadic internal septa. Cystic formations had slightly distinct signal, native image in T1 weighing displayed hypersensitive contents on the right side. It could be a case of chronic post haemorrhagic changes. Postcontrastly the collections were without amplification of signal intensity. On the left side postcontrastly there was present a moderate reinforcement of capsule of fluid collection. In diffuse weighing the lesions were without marks of diffusion restriction. Axillary lymphatic nodes were of physiological size, the displayed pulmonary parenchyma was without inflammatory and focal changes, without mediastinal and hilar lymphadenopathy, the pleural cavities without effusion, the pleura was without hypertrophy, the recorded skeleton was without traumatic change.

**Fig. 2:**
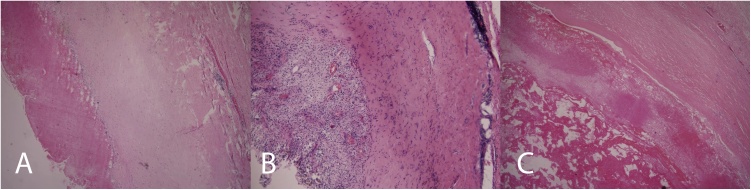
(a): Histological preparation – Haematoxilin and eosin, magnification 40×. Lesion wall is formed by collagenous, hyanilinised and vascularised connective tissue with predominately perivascular nonspecific chronic inflammatory cellulation. (b): Histological preparation - Haematoxilin and eosin, magnification 100×. Internal surface of lesion is lined with layer of fibrin and nonspecific granulation tissue with focally accentuated siderophagous and giant-cell reaction. (c): Histological preparation – Haemotoxilin and eosin, magnification 40×. In lumen of pseudocyst there are remains of blood clots with presence of fibrinous or fibrinoid substances with dispersive admixture of siderophages, lymphocytes, neutrofiles and giant polynuclear cells.

Owing to progressing swelling and increasing difficulties a surgical resection was indicated in the female patient. She was operated on under general anaesthesia, and a resection of the encapsulated collections of fluid was implemented bilaterally (Fig. 1(d)), two Redon drains were introduced. In the left collection serous fluid was present, on the right side also serous fluid with admixture of old blood was present. A histological examination of cystic collections proved that it concerned pseudocystic lesions with relation to subscapular bursa without marks of malignancy. Their walls were created by collagenous, hyalinised and vascularised connective tissue with predominately perivascular nonspecific chronic inflammatory cellulation (Fig. 2(a)), the internal surface of which was lined by a layer of fibrin and by a nonspecific granulation tissue with a focally accentuated xanthogranulomatous, siderophagous and giant-cell reaction without epithelium (Fig. 2(b)). In the lumen of the cysts there were remnants of blood clots with fibrinous or fibrinoid substances with dispersive admixture of siderophages, lymphocytes, neutrophils and giant polynuclear cells (Fig. 2(c)). The proof of amyloid by Congo red was negative. On the lesion periphery soft-tissue structures were caught, including striated muscularis. The drains were removed the 10th postoperative day due to higher production, the surgical wounds were healed-up per primam intentionem. After the operation the female patient had a full range of movements and was without trouble and pains.

## 3 Discussion

Scapulothoracic articulation has several bursae which enable a sliding movement of scapulothoracic joint. The two major anatomic bursae are the supraserratus bursa and infraserratus bursa. The supraserratus bursa lies between the musculus serratus anterior and musculus subscapularis, the second bursa is between the musculus serratus anterior and thoracic wall [1]. There are four adventitious bursae of the scapulothoracic articulation, the bursae minores. Two bursae are located on the superomedial angle of the scapula, one is on the inferior angle of the scapula and the fourth bursa is situated on the back of the scapula under the musculus trapezius [3]. In our female patient the infraserratus bursa was affected. Bursae can be inflamed secondarily and so a bursitis with bursa filling-in with liquid content arises. The secondary inflammation is most frequently caused by trauma, sport, or by repeated mechanical movements. Soft tissues of the scapulothoracic joint will be damaged by a direct or indirect trauma. In sports like golf, tennis, swimming or in throwing, the syndrome of overuse can appear [4] and also in work which requires repeating or standing movements of the scapula against the posterior thoracic wall [3]. Our patient worked as a rehabilitation worker, her work contributed to her injury. Further causes of scapulothoracic bursitis include glenohumeral joint dysfunction, bony abnormalities, muscular atrophy or fibrosis and idiopathic causes [2]. Muscular atrophy around the scapula and thoracic wall can increase the friction between the musculus serratus anterior and thoracic wall, which leads to the genesis of bursitis. Bony deviations of the thoracic wall after fractures of ribs may aggravate the state of already existing bursitis [3]. Fujikava et al. describe the genesis of scapulothoracic bursitis after a secondary formation of bursa owing to thoracoplasty at a patient with pulmonary tuberculosis [5].

A clinical examination of patients with bursitis should begin with a physical examination accompanied by taking a history. Scapulothoracic bursitis in patients manifests itself by pain, swelling, without reddening. The pain is usually escalated at increasing activity. By inspection a cystic resistance between musculus serratus anterior and thoracic wall, well defined and worse movable will be discovered. In some patients a crepitus at scapula movement can be audible and tangible [2].

In differential diagnostics of the thoracic wall focus in the scapulothoracic region it can be a case of abscess, haematoma, elastofibroma, sarcoma, malignant fibrous histiocytoma or liposarcoma. In differentiation between the scapulothoracic bursitis and tumours of soft tissues of the thoracic wall imaging examinations like computer tomography and magnetic resonance are useful [4]. The Magnetic resonance is the best tool for revelation of pathology of soft tissues. Ultrasonography performed with high precision by an experienced doctor can also be a primary imaging alternative [3]. In magnetic resonance imaging the cystic lesions are shown between musculus serratus anterior and thoracic wall, the high intensities of signal on T1 and of T2 weighted images and the level of fluid on the T2 weighted images are findings testifying for scapulothoracic bursitis [6].

The therapy of bursitis can be nonoperative or operative. Conservative treatment includes exercises of the arms, anti-inflammatory drugs, intracystic injections of long-term-acting corticoids or ethanol [4]. Also local hot compresses, relaxation utilization of ultrasound stimulation of nerves [3] belong among further ways of conservative therapy. Ciullo and Jones [7] used ionophoresis for therapy of symptomatic scapulothoracic bursitis.

The surgical therapy of bursitis is indicated, if a conservative treatment was shown as unsuccessful [4,2]. Above all it concerns patients with pronounced painfulness, excessive friction between musculus serratus anterior and thoracic wall or with dysfunction of glenohumeral joint [6]. The possibilities of operative treatment include open bursectomy, partial scapulectomy and resection of bursa by arthroscopic technique of mini-invasive surgery [4,2]. To the complications connected with the arthroscopic or open bursectomy we classify pneumothorax, haematoma, damage of nervus scapularis a recurrence of bursitis [2].

The surgical treatment of scapulothoracic bursitis has good results, and the patients are able to return into employment, alternatively to sport activities [2].

## 4 Conclusion

This case report describes rare bilateral cystic lesions of thoracic wall, which were a manifestation of scapulothoracic bursitis. Radical surgical therapy represented an effective treatment strategy with good postoperative results.

## Conflicts of interest

No potential conflict of interest was reported by authors.

## Sources of funding

This case report had no involvement sponsors.

## Ethical approval

This case report is exempt from ethical approval by our institution.

## Consent

Written informed consent was obtained from the patient for publication of this case report and accompanying images. A copy of the written consent is available for review by the Editor-in-Chief of this journal on request

## Author contribution

Dzian - study concept, data collection, surgical therapy for this patient

Skaličanová - study design, writing the paper

Fučela - surgical therapy for this patient

Malík - data collection

Mičák - analysis and interpretation of histological preparation

## Registration of research studies

None.

## Guarantor

Anton Dzian, M.D.

## Provenance and peer review

Not commissioned externally peer reviewed.
